# Molecular mechanism(s) of regulation(s) of c-MET/HGF signaling in head and neck cancer

**DOI:** 10.1186/s12943-022-01503-1

**Published:** 2022-01-26

**Authors:** Sibi Raj, Kavindra Kumar Kesari, Arun Kumar, Brijesh Rathi, Ashok Sharma, Piyush Kumar Gupta, Saurabh Kumar Jha, Niraj Kumar Jha, Petr Slama, Shubhadeep Roychoudhury, Dhruv Kumar

**Affiliations:** 1 Amity Institute of Molecular and Stem Cell Research (AIMMSCR), Amity University Uttar Pradesh, Noida, 201313 India; 2 Department of Applied Physics, School of Science, Aalto University, 00076 Espoo, Finland; 3 Mahavir Cancer Institute & Research Center, Phulwarisharif, Patna, Bihar 801505 India; 4 Laboratory for Translational Chemistry and Drug Discovery, Department of Chemistry, Hansraj College, University of Delhi, Delhi, India; 5 Department of Biochemistry, All India Institute of Medical Science (AIIMS), Ansari Nagar, New Delhi, Bharat 110029 India; 6 Department of Life Sciences, School of Basic Sciences and Research, Sharda University, Greater Noida, 201310 India; 7 Department of Biotechnology, School of Engineering and Technology (SET), Sharda University, Greater Noida, UP 201310 India; 8 Department of Animal Morphology, Physiology and Genetics, Faculty of AgriSciences, Mendel University in Brno, Brno, Czech Republic; 9 Department of Life Science and Bioinformatics, Assam University, Silchar, 788011 India

**Keywords:** Head and neck squamous cell carcinoma, C-MET, EGFR, Hepatocyte growth factor, Chemoresistance, Monoclonal antibody

## Abstract

Head and neck cancer is the sixth most common cancer across the globe. This is generally associated with tobacco and alcohol consumption. Cancer in the pharynx majorly arises through human papillomavirus (HPV) infection, thus classifying head and neck squamous cell carcinoma (HNSCC) into HPV-positive and HPV-negative HNSCCs. Aberrant, mesenchymal-epithelial transition factor (c-MET) signal transduction favors HNSCC progression by stimulating proliferation, motility, invasiveness, morphogenesis, and angiogenesis. c-MET upregulation can be found in the majority of head and neck squamous cell carcinomas. c-MET pathway acts on several downstream effectors including phospholipase C gamma (PLCγ), cellular Src kinase (c-Src), phosphotidylinsitol-3-OH kinase (PI3K), alpha serine/threonine-protein kinase (Akt), mitogen-activated protein kinase (MAPK), and wingless-related integration site (Wnt) pathways. c-MET also establishes a crosstalk pathway with epidermal growth factor receptor (EGFR) and contributes towards chemoresistance in HNSCC. In recent years, the signaling communications of c-MET/HGF in metabolic dysregulation, tumor-microenvironment and immune modulation in HNSCC have emerged. Several clinical trials have been established against c-MET/ hepatocyte growth factor (HGF) signaling network to bring up targeted and effective therapeutic strategies against HNSCC. In this review, we discuss the molecular mechanism(s) and current understanding of c-MET/HGF signaling and its effect on HNSCC.

**Graphical abstract:**

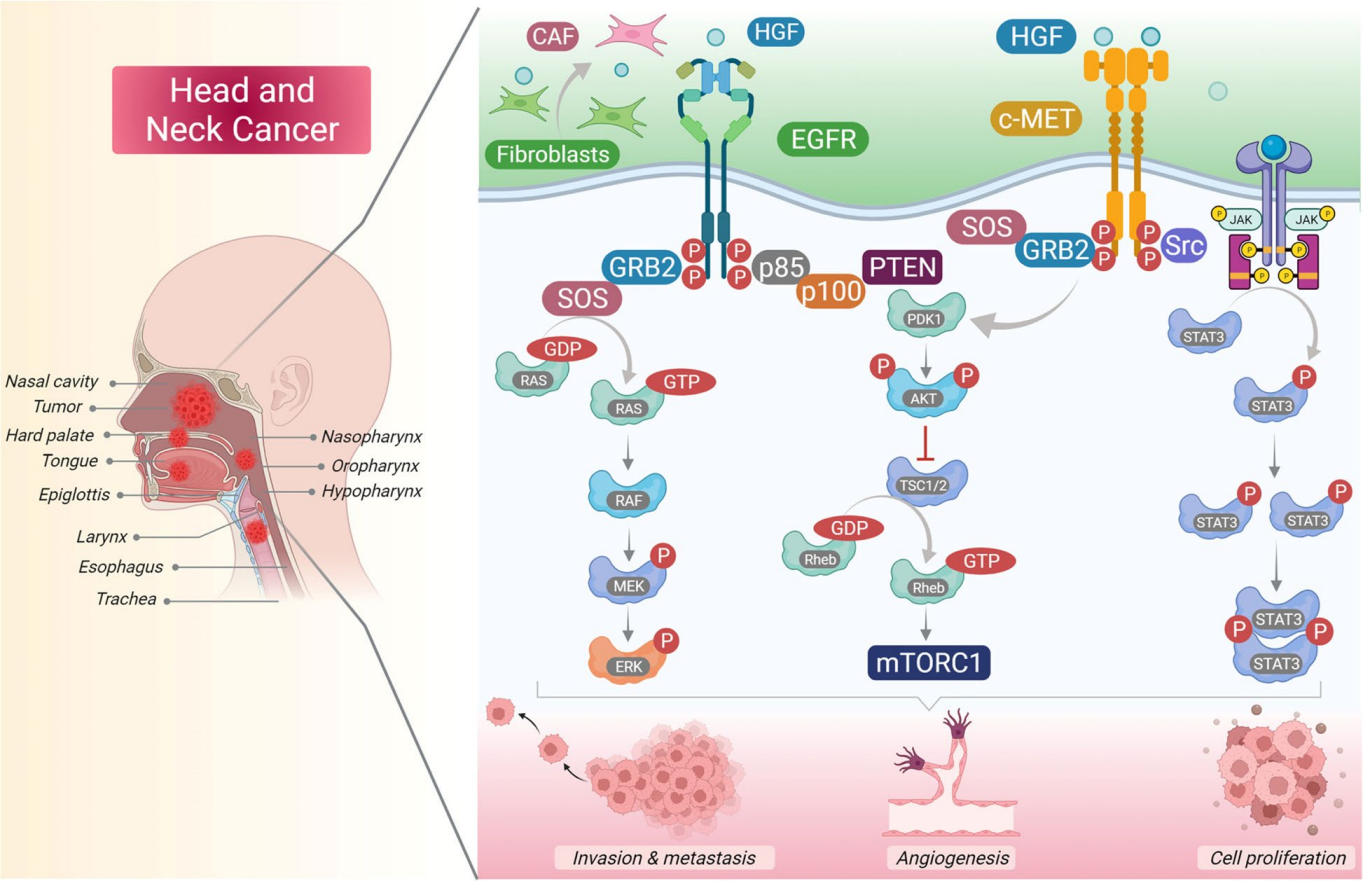

## Background

The process of development is a journey beginning through embryogenesis, where cells bud off from developing tissues and move outwards to form shape and patterns that make up the complex architecture of a living organism. Certain cellular activities such as motility, survival and proliferation follow invasive growth pattern which is mostly triggered by extracellular stimuli. This may also regulates the activity of several transcription factors to modulate the expression of proteins such as cytoskeletal, cell-cell junctional components, cell cycle regulators and anti-apoptotic effectors. Mesenchymal-epithelial transition factor (c-MET) function has been reported to be vital in various morphogenetic events in both embryonic and adult stages [1]. c-MET is a tyrosine kinase receptor which belongs to the MET (MNNG HOS transforming gene) family. It is usually found to be expressed on the surface of various epithelial cells. (Hepatocyte growth factor/scatter factor (HGF/SF) is the common ligand to c-MET receptor [2]. HGF was first reported structurally similar to plasminogen in early 1980s, which was further characterized by a new substance responsible for liver regeneration in the serum of partially hepatectomized rats [3]. The term scatter factor was later introduced independently in 1985 by Stoker and co-workers, as they discovered a protein interrupting the intracellular junctions of epithelial cells thereby promoting their migration in a paracrine manner and promoting an invasive phenotype [4]. But closer analysis into scattering factor through sequence analysis and complementary deoxyribonucleic acid (cDNA) cloning concluded that HGF and scattering factor are indeed indistinguishable from each other.

Cooper et al. were the first to identify the MET oncogene in 1984. They chemically transformed human osteosarcoma cell line by transfection analysis in NIH/3 T3 cells and mapped the MET oncogene [5]. MET oncogene was mapped to chromosome bands 7q21–31 and showed a genetic translocation between two distinct loci, the MET proto-oncogene at chromosome 7 and translocator promoter region (TPR) at chromosome 1. This was later detected on the cell surface upon analyzing the translational product of 21-exon MET proto-oncogene and was classified as a growth factor receptor of the tyrosine kinase family. The product of MET proto-oncogene acts as a transmembrane tyrosine kinase receptor for HGF [6]. The receptor is mainly located on melanocytes, endothelial cells, and epithelial tissues, including those of the liver, gastrointestinal tract, kidney and other organs.

**Fig. 1:**
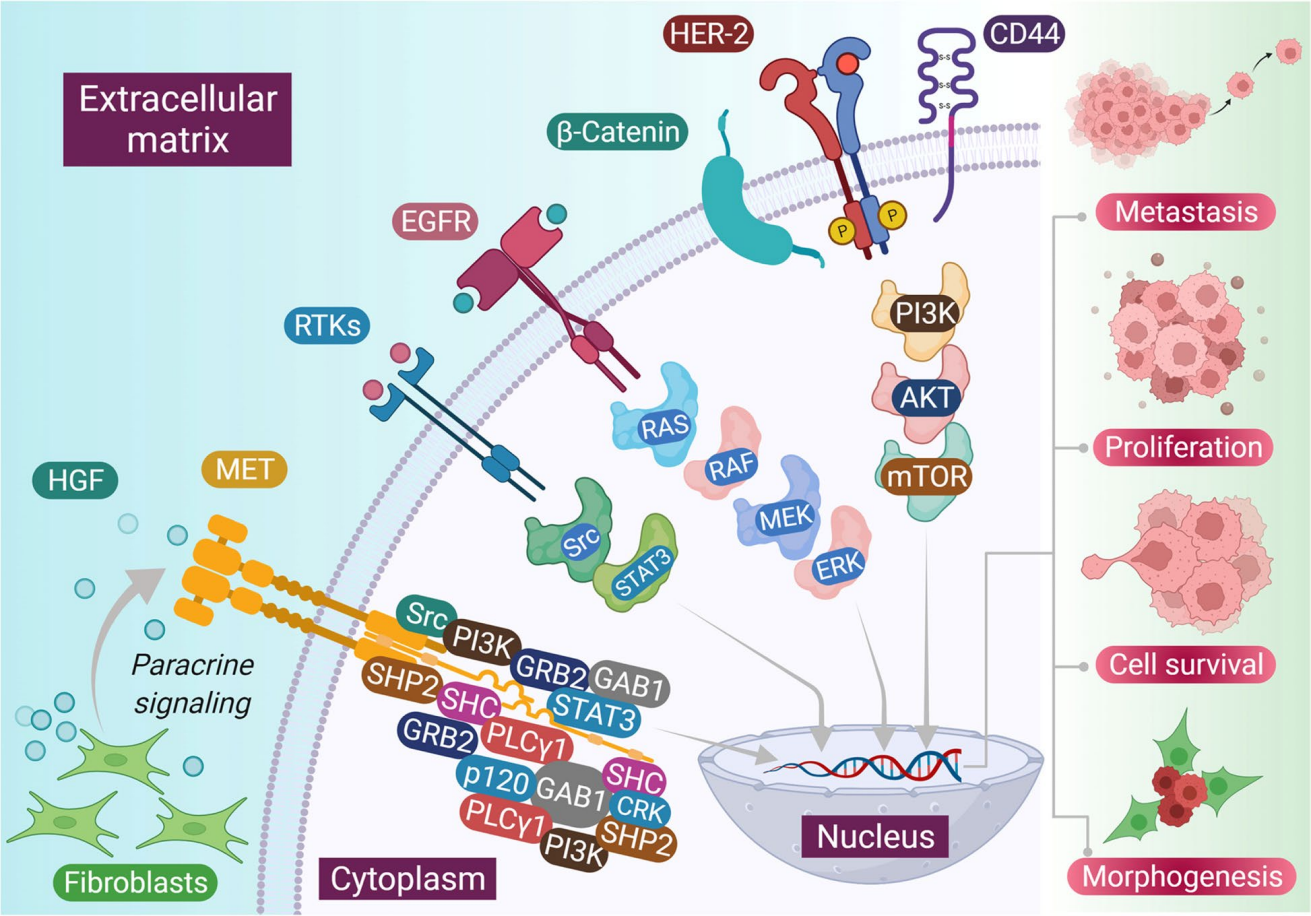
Different downstream signaling pathways activated through c-MET and its interactive other membrane receptors. HGF released through tumor associated fibroblast cells in microenvironment binds to c-MET and activates it through autophosphorylation of tyrosines Y1234 and Y1235 within the activation loop of the kinase domain and subsequent phosphorylation of tyrosines Y1349 and Y1356 near the -COOH terminus. Major adapter proteins and direct kinase substrates activated downstream in the c-MET pathway include growth factor receptor-bound protein 2 (GRB2), Grb2-associated adaptor protein 1 (GAB1), phosphatidylinositol 3-kinase (PI3K), son of sevenless (SOS), rat sarcoma oncogene homolog (RAS), mitogen-activated protein kinase (MAPK), signal transducer and activator of transcription 3/5 (STAT 3/5), SRC, SRC homology protein tyrosine phosphatase 2 (SHP2), SRC homology domain c-terminal adaptor homolog (SHC), phospholipase c-γ (PLC), Ras-related C3 botulinum toxin substrate 1 (RAC1), p21-activated kinase (PAK), focal adhesion kinase (FAK), AKT, and mammalian target of rapamycin (mTOR). Crosstalk between c-MET and various membrane protein partners, including the epidermal growth factor receptor (EGFR), the plexin B family, α6β4 integrin, and CD44, results in additional signaling response modulation

HGF activates c-MET receptor in a paracrine manner by exerting its pleotropic effects through controlling several transmission cascades such as the mitogen-activated protein kinase (MAPK), phosphatidylinositol-3 kinase (PI3K)/AKT, and Janus kinase/signal transducer and activator of transcription (JAK/STAT) pathways [7]. c-MET networking has established crosstalk with various other signaling molecules that functions in certain physiological processes (Fig. 1). Apart from mitogenic, motogenic, and morphogenic properties, c-MET also protects against apoptosis by its interaction with PI3K/AKT (phosphatidylinositol 3-kinase/protein kinase-B) pathway. Although, co-expression of both c-MET and HGF were detected at various events of wound closure, tissue regeneration, and embryogenesis [8]. However, all these key physiological processes can be exploited during cancer proliferation, invasion and metastasis. Cancer is known for its ability to evade the cell cycle regulatory events and involves abnormalities in extracellular signaling, transcellular transduction, and intracellular networking. An aberrant HGF/c-MET signaling can lead to uncontrolled proliferation, motility, invasiveness, and angiogenesis and can play an essential role in the development, progression and survival of cancer including head and neck squamous cell carcinoma (HNSCC) [9].

HNSCCs originate from the mucosal epithelium in the oral cavity, pharynx and larynx and are most common malignancies that arise in head and neck. This has been correlated to lifestyle factors such as tobacco smoking, excessive alcohol consumption, or both. Tobacco related carcinogens undergo metabolic activation with the help of detoxifying enzymes and pathways promoting excretion [10]. These reactive molecules can also form DNA adducts, which under repair impairment can lead to mutations and other genetic abnormalities. Human papillomavirus (HPV) has been associated with tumors that arise in the oropharynx especially the HPV-16 and to a lesser extent, HPV-18 and other strains [11]. Since, there are FDA-approved vaccines available against HPV-16 and HPV-18, there are high chances to prevent HPV related HNSCCs. The HNSCCs originating from oral cavity and larynx are largely associated with smoking and are also referred to as HPV negative HNSCCs [12, 13]. There is an increased need to identify molecular biomarkers that can be used to predict progression of HNSCC for better survival and reveal new targets to develop new therapeutic agents. Molecular biomarkers such as CD44, CD133 and ALDH1 have been extensively validated for their prognostic significance for HNSCC cancer stem cells (CSC) [14, 15]. The Cancer Genome Atlas (TCGA) contains enough data that validates on copy number alterations (CNA), mutational profiles, mRNA expression and microRNA (miRNA) expression. In addition to genetic alterations, epigenetic changes also play an important role in driving HNSCC oncogenesis. Although, DNA hypomethylation, hypermethylation, and down regulation of tumor suppressor genes (TSGs) have been recognized as a major factor for HNSCC progression. Several signaling pathways have their role in HNSCC progression. Additionally, EGFR is overexpressed in 80–90% of HNSCC tumors and has been associated with poor and progression free survival [16]. Also, crosstalk with other signaling pathways such as transforming growth factor-beta (TGF-β) has led to carcinogenesis and resistance to targeted therapies in human lung, pancreatic, and breast tumor xenografts [17].

There is no such screening strategy available that proves effective rather careful physical examination that has been a primary approach for early detection. A proportion of pre-malignant lesions are present as either leucoplakia (white patches) or erythroplakia (red patches) which can progress to advanced stage of HNSCC. HNSCC in the oral cavity is primarily treated through surgical resection followed by adjuvant radiation or chemotherapy plus radiation depending on the disease stage [18]. However, depending on the disease stage cancer in the larynx and pharynx is primarily treated through chemo radiotherapy (CRT). HPV positive HNSCC has shown more favorable prognosis than the HPV negative HNSCC which can also lessen the therapeutic dose of both radiation and chemotherapy in HPV positive disease treatment. Indeed, cetuximab which is an epidermal growth factor receptor (EGFR) monoclonal antibody, has been approved by FDA as a radiation-sensitizer, alone or in combination with chemotherapy for patients with recurrent or metastatic diseases. Moreover, cisplatin has been recognized as another radiosensitizer in HPV associated disease. Pembrolizumab and nivolumab which have also been in a category of immune checkpoint inhibitors are approved by FDA as a first line therapy in patients having cisplatin insensitive recurrent or metastatic HNSCC [19–21]. Targeting c-MET receptor provide a strong rationale for exploring the therapeutic potential of specific receptor antagonists. Although till now no clinically relevant overall survival benefit has been reported by selective inhibition in any cancer types.

### c-MET

c-MET is a receptor tyrosine kinase primarily located on melanocytes, endothelial cells, and epithelial tissues, including those of the liver, gastrointestinal tract, kidney and other organs. In normal cells, the primary MET transcript produces a peptide of 150 kDa, which is partially glycosylated to produce a precursor protein of 170 kDa [22]. The receptor was originally isolated from a human gastric tumor cell line and contains a α chain of 50 kDa and a β chain of 145 kDa which are linked with disulphide bridges in a transmembrane αβ complex of 190 kDa. A precursor protein of 170 kDa is post-translationally modified via glycosylation and cleavage to give rise to a mature heterodimer [23]. The smaller α-subunit lies on the extracellular surface while the single-pass β chain forms a backbone for three main regions of c-MET. The extracellular region of c-MET has high affinity toward HGF while the single transmembrane segment and the cytoplasmic tyrosine kinase domain is edged by juxtamembrane and carboxy-terminal sequences. There are three domain types present at the extracellular portion. SEMA domain containing 500 amino acids relies on the entire α and β subunits [24]. Conserved sequences of c-MET receptor have structural homology to large family of ligand-receptor pairs containing semaphorins and plexins. Semaphorins are large family of secreted proteins that helps in the cell dissociation and repulsive guidance events in situations like cell scattering during neural development. Below SEMA domain is the PSI domain which is common to plexins, semaphorins and integrins [25]. It is attached to the transmembrane helix by four IPT (immunoglobulin-like, plexins, transcription factors) domains. HGF can bind to two sites, one which is located on the SEMA domain having low-affinity and high specificity contacts, the other located in the IPT domain having low-specificity and high affinity contacts [26]. The intracellular domain has three portions: a juxtamembrane sequence that downregulates kinase activity which is followed by phosphorylation of Ser975 which is a catalytic region that promotes kinase activity leading to transphosphorylation of Tyr1234 and Tyr1235. Upon ligand binding dimerization of receptors takes place which is triggered by phosphorylation of tyrosine residues located in the kinase domains. This may lead to autophosphorylation of a bidentate docking site in the carboxy-terminal cytoplasmic tail (tyrosine residues Tyr1349 and Tyr1356). MET as a proto-oncogene has a total length of 125,982 bp, and is located in the chromosome 7 locus 7q31. HGF is a member of plasminogen related growth factor family and also referred to as PRGF-1 [27]. The gene encoding HGF spans 70 kb on chromosome 7q21.1 is initially in the form of pro-HGF, which is further cleaved by a protease to mature HGF [28].

### c-MET signaling

HGF is the main ligand to c-MET receptor to activate its biological function. HGF is produced as a single-chain inert precursor. Cleavage by extracellular proteases converts the precursor into a two-chain functional heterodimer. HGF is majorly distributed in the extracellular matrix of most tissues in its inactive form where it is cleaved by heparin-like proteoglycans to its mature form [29]. HGF largely originate from mesenchymal cells which acts in a paracrine manner on epithelial cells that express the c-MET receptor. Several cytokines like interleukin 1, 6, tumor necrosis factor-α and transforming growth factor-β induces transcriptional upregulation of both HGF residing in fibroblasts and macrophages and c-MET residing in epithelial cells [30]. The tumor stroma elevates the expression of proteases such as plasminogen activation system and matriptase that are involved in the activation of pro-HGF. Thus, HGF is activated and over produced for biological functions by controlled combination of transcriptional and post transcriptional regulation, which also eventually leads to optimal MET activation on target cells. This can also be a huge part of general mechanism of physiological defense to tissue damage. Upon HGF binding c-MET gets activated through kinase activity which is followed by receptor dimerization and trans-phosphorylation of two catalytic tyrosine residues Tyr1234 and Tyr1235 within the kinase activation region [31]. This is followed by further phosphorylation of two additional docking tyrosine residues in the carboxy terminal tail (Tyr1349 and Tyr1356) which on activation can act as degenerate motif to recruit several other subsequent signaling molecules. MET acts as a substrate for several protein tyrosine phosphatases, including the receptor PTPs density enhanced phosphatase 1 (DEP 1) and leukocyte common antigen related (LAR) and the non-receptor PTPs, PTP1B and T cell PTP [32]. These phosphatases might interrupt the c-MET signals by promoting dephosphorylation of catalytic or docking tyrosines. There are many scaffold proteins that are associated along with c-MET receptor and leads to activation of downstream signal transduction pathways that include mitogen-activated protein kinase (MAPK) cascades, extracellular signal-regulated kinase 1 (ERK1) and ERK2, Jun amino-terminal kinases (JNKs) and p38, the phosphoinositide 3-kinase–Akt (PI3K–AKT) axis, signal transducer and activator of transcription proteins (STATs), and the nuclear factor-κb inhibitor-α (iκbα)–nuclear factor-κb (NF-κb) complex [33]. The C-terminal tail of c-MET binds to numerous Src homology 2 domain containing effectors, such as PI3-K, the non-receptor kinase Src, the growth factor receptor bound protein 2 (GRB2) and SH2 domain-containing transforming protein (SHC) adaptors SHP2 (also known as PTPN11; an upstream activator of Src and ras), phospholipase Cγ1 (PlCγ1) and the transcription factor STAT3 [34]. c-MET receptor is also associated with GRB2-associated binding protein 1 (GAB1) phosphorylation and provides extra binding sites for other adaptor proteins. GAB1 is associated with c-MET receptor via 13 amino acid MET binding sites and indirectly through MET-bound GRB2 [35]. Several transducer proteins participate in the c-MET receptor signaling cascades majorly with the interactions with GAB1 scaffolding adaptor. CD44 has been recognized as another protein that interacts with c-MET in normal and transformed epithelial cells.

Activation of HGF/c-MET signaling requires phosphorylation of several elements in the c-MET receptor. Once HGF binds to the c-MET receptor autophosphosphorylation of several tyrosine residues takes place in the intracellular region. The activation of intracellular kinase activity of c-MET takes place via phosphorylation of Y1230, Y1234 and Y1235 [36]. Y1313 is an important residue that associates with PI3K and it can be phosphorylated in response to HGF binding. Phosphorylation of tyrosine residues Y1349 and Y1356 present in the C-terminus of c-MET receptor activates the multisubstrate docking site which activates further signaling pathways. The multisubstrate docking sites have domains for Src homology-2 domains, phosphotyrosine binding (PTB) domains, and MET binding domains (MBD) of signal transducers and adapter proteins [37]. Receptors containing these amino acid sequences can activate further cellular responses which are similar to c-MET interactions. The juxta membrane domain contains the Y1003 residue which binds to proteins such as c-Cbl, the protein responsible for ubiquitination of c-MET thereby its activation. Cbl recruits the endophilin-CIN85 complex and regulates c-MET internalization, suggesting its role in HGF/c-MET signaling regulation [38].

### Head and neck cancer

HNSCC is the sixth most common cancer worldwide [39, 40]. It develops from the mucosal epithelium in the oral cavity, pharynx, larynx and are the most common malignancies that takes place in the head and neck [41]. The common practices or risk factors that has been associated with HNSCC are smoking, excessive alcohol consumption, or both [42]. Cancer causing effects of tobacco relies on the presence of carcinogens such as polycyclic aromatic hydrocarbons (PAHs), benzo(a)pyrene, nitrosamines, 4-(methyl nitrosamine)-1-(3-pyridyl)-1-butanone and N-nitosonornicotine [43]. These carcinogens undergo metabolic activation along with detoxification enzymes and pathways promoting excretion. Tobacco products generally promote genetic changes leading to metabolic activation and improper DNA repair mechanisms [44]. While nicotine itself is not considered to be carcinogenic, each cigarette contains a mixture of carcinogens, including a small dose of polycyclic aromatic hydrocarbons (PAHs) and 4-(methylnitrosamino)-1-(3-pyridyl)-1-butanone (NNK) among other lung carcinogens, tumor promoters, and co-carcinogens. Carcinogens such as NNK and PAHs require metabolic activation to exert their carcinogenic effects; there are competing detoxification pathways, and the balance between metabolic activation and detoxification differs among individuals and will affect cancer risk. The metabolic activation leads to the activation of DNA adducts which if escapes the repair mechanism can result in miscoding and permanent mutation. If this mutation takes place in critical region of an oncogene or tumor suppressor gene, it can lead to cancer. Excessive alcohol consumption leads it to act as a carcinogen solvent subsequently increasing the exposure of epithelial cells to these substances. Also, alcohol is metabolised to acetaldehyde which is reported to form DNA adducts [45].

The increased cases of malignancies in oropharynx have been associated with prior infection with oncogenic strains of HPV, primarily of HPV-16 and HPV-18 [12, 46]. HPV infection is said to be mainly transmitted via oral sex and the incidence of HPV-positive HNSCC continues to rise among population that are not vaccinated against HPV prior to its exposure. HPV infection causing early HNSCC mostly arises from deep crypts in the palatine and lingual tonsils. HPV-16 is the primary causative type, whereas other HPV types including HPV-18, HPV-31, HPV-33 and HPV-52 are also detected in small amounts in HNSCC patients. HPV-16 is a small double stranded circular DNA virus having a genome of size 8 kb [13, 47, 48].

HNSCC is also defined by genetic instability with frequent loss or gain of chromosomal regions. 9p21 loss takes place at advanced stages of HNSCC [49, 50]. This region includes tumor suppressor genes, CDKN2A which encodes for CDK4 and CDK6 inhibitor p16^INK4A^ and ARF encoding p14 which is a stabilizer of p53. The accumulation of altered key tumor suppressor genes such as TP53 and CDKN2A, which encode p53 and p16INK4A, respectively is associated with the onset, progression and poor prognosis of HPV-negative HNSCC. Progression of HNSCC from hyperplasia to dysplasia is marked by the loss of 3p21 and 17p13. Further transition into tumor involves the loss of 11q13, 13q21 and 14q32 [51]. These evidences suggest the involvement of strong genetic alterations in HNSCC progression into invasive carcinoma. Epigenetic analysis of the c-MET promoter identified significant loss of DNA methylation in CTCs which correlated with overexpression of c-MET and increased expression of HGF. Six specific CpG sites of c-MET promoter demethylation was identified. CTCs show significantly increased tumorigenicity and metastatic potential in a novel orthotopic syngeneic model of metastatic cancer [52].

Patients with inherited genetic diseases such as Fanconi anemia having impaired DNA repair are reported to have 500–700-fold increased risk of developing HNSCC, primarily in the oral cavity. Several studies have reported aberrant signaling pathways playing a valid role in HNSCC. Molecular targeting of EGFR with monoclonal antibodies such as cetuximab is an FDA-approved strategy for inhibiting EGFR signaling in HNSCC [53]. Increased expression of receptor tyrosine kinases such as HER-2 and MET which can lead to resistance towards EGFR mediated therapy against HNSCC. Increased expression of cytokine IL-6 and its receptor also shows poor prognosis of HNSCC [54]. PI3K-AKT-mTOR signaling pathway has been reported to be the most frequently altered oncogenic pathway and is genetically altered in most HNSCC tumors. Electronic cigarettes have a major role in HNSCC which has to be made evident in coming years due to its high usage [55]. Some other risk factors include ageing, poor oral hygiene and improper diet intake without vegetables. There have been no effective screening strategies rather than careful physical observation, which remains as the primary approach for early detection. Majority of the oral initial stage lesions are present in the form of white patches known as leucoplakia or in red patches known as erythroplakia. This heterogeneous nature of HNSCC depends on cell origin on anatomical location and etiological agent. HNSCC in the oral cavity and larynx are usually treated with surgical removal of affected tissues which is then followed by adjuvant radiation or chemotherapy along with radiation known as chemoradiotherapy (CRT) [56]. Cancers that occur in the pharynx or larynx are primarily treated through CRT.

Several biomarkers associated with HNSCC prognosis has been validated which includes CD44, CD133 and ALDH1. CD44 is a cell surface receptor for hyaluronic acid and matrix metalloproteinases (MMPs) and is associated with intracellular interactions and cell migration [57]. Increased level of CD44 enables self-renewal and is also associated with metastasis and poor prognosis. Similarly, high levels of membrane-spanning protein CD133 is associated with HNSCC invasiveness and metastasis [58]. ALDH is a marker for both normal stem cells and CSCs and is an intracellular enzyme that converts retinol into retinoic acid, and detoxifies the cellular system [59]. Also, stem cell markers such as OCT3, OCT4, SOX2 and NANOG shows an effective way to correlate the analysis of tumor grade in oral tumors.

Several aberrant signaling pathways also equally contribute in HNSCC progression. EGFR is the commonly expressed receptor in among 80–90% of HNSCC tumors and has also been associated with poor overall and progression free survival. Over expression of other tyrosine kinase receptors such as HER-2, c-MET has also been widely reported in the case of HNSCC enabling resistance towards receptor targeting monoclonal antibody therapies against HNSCC [60]. Studies reported that MET gene (N375S) polymorphism has the binding affinity for HER-2 and enables the interaction with HER-2 in a ligand independent fashion. This interaction transduces potent proliferative and pro-metastatic characteristics in HNSCC through HER-2 signaling and is associated with poor prognosis [60]. PI3K-AKT-mTOR pathway has been the common drive for HNSCC development as it is the most altered oncogenic pathway in HNSCC [61]. This pathway is mostly genetically altered by the presence of mutation and gene amplification in PI3KCA. Loss of PTEN function takes place in HNSCC patients, which is a negative regulator PI3K-AKT signaling pathway [62]. Another pathway which is hyperactivated in HNSCC is STAT3 and is correlated with poor prognosis of HNSCC. STAT3 hyperactivation has been correlated with mutations in the gene encoding protein tyrosine phosphate receptors (PTPRs) PTPRT and PTPRD which takes place frequently in HNSCC [63]. STAT3 hyperactivation promotes cellular proliferation, survival as well as immunosuppression by promoting VEGF, IL-6, IL-10 and TGF-β. Alterations in Wnt/β-Catenin pathway also contribute to the survival of HNSCC. RAS-MAPK pathway is yet another mutated pathway to have been found in HNSCC tumors.

HNSCC is majorly a cancer of adults with a median age of diagnosis of 66 years for HPV negative and 53 years for HPV positive HNSCC. Men have reportedly been at higher risk for developing HNSCC as compared to the female population [64]. HNSCC diagnosis is done through biopsy of primary tumor depending on its location. The tumor is accessed through the cup forceps, incisional or excisional biopsy. Fine needle aspiration strategies are preferred for suspicious cervical neck mass. The diagnosis of HNSCC is usually performed through routine based histopathological analysis via hematoxylin and eosin staining [65]. However, immunohistochemistry-based studies are performed in case of poorly differentiated or basaloid tumors to confirm the epithelial origin. HPV testing is necessary for all the oropharyngeal or unknown primary tumors to determine modern staging and prognosis. HPV determination is usually done through E6 and E7 mRNA detection or the cell cycle protein p16^INK4An^ [66, 67]. The primary curative strategy for locally confined HNSCC are resection, radiation and systemic therapy. Patients at initial stages with small primary cancer can be treated with single modality intervention via resection or radiation. Surgery is the main approach for oral cavity cancers whereas radiation is the common approach for pharyngeal and laryngeal cancers.

### c-MET and immune response

Cancer is a smart disease which finds quick alternatives against therapeutic strategies to evade apoptosis and continue its progression in the body. One of such smart evading responses include the ability of cancer cells to evade the anti-tumor immune responses. However, inhibition of checkpoint proteins has established ways to overcome immunosuppression and evolved cancer immunotherapy [68]. Studies have suggested the association of c-MET expression with expression of immunoregulatory molecules such as programmed cell death ligand (PDL-1) and indoleamine-2,3-dioxygenase (IDO) in cancer cells [69]. This promises c-MET to be an effective target for immunotherapy as there are clinically available targets against c-MET. Programmed death 1 (PD-1) is a protein molecule expressed on the surface of T-cells in response to antigen stimulation. Upon the clearance of antigen PD-1 expressed in low levels, but in the case of cancer PD-1 remains highly expressed on T-cells. To prevent the excessive antigen stimulation response, cancer cells suppress the T-cell activity by binding of PD-1 to ligand on cancer cells such as PDL-1. PDL-1 expression has been positively correlated with MET activation in lung cancer cells. Thus, studies suggest c-MET not only as an anti-tumor target but also a good target for immunotherapy.

c-MET can either act as an immunosuppressive agent by negatively affecting dendritic cells (DC) and T lymphocytes or can stimulate immune response by promoting recruitment of DC, B-cells and T-lymphocytes [70]. Overexpression of MET in cancer cells can make c-MET to be recognized as a tumor associated antigen (TAA) by CD8 cytotoxic T cells which can trigger immune system activation [71]. The widely studied role of c-MET in immune system is associated with DC. DC are involved in TAA and induce the activation of CD4^+^ T-regulatory cells which controls the cytotoxic T cells. Studies have shown HGF possibly stimulating this function and suggesting its positive role in immunotherapy [72–74]. Apart from role with antigen presenting cells, c-MET has also been identified in granulocytes. Deletion of MET in neutrophils amplified the tumor growth and metastasis. In clinical studies, deletion of MET in neutrophils has been associated with poor neutrophil infiltration in both primary and distant metastases [75]. The important aspect to see while treating tumor patients with MET inhibitor is that tumor derived tumor necrosis factor-α (TNF-α) or any other inflammatory stimuli enhances the MET activation in human neutrophils and leads to their transmigration across an activated endothelium and releases free radicals, which can inhibit the cancer cell growth [76]. Therefore, when treated with MET inhibitors, it can actually help in cancer progression in patient in such cases. Thus, these studies suggest the complex role of c-MET in cancer as it can act as a tumor suppressor or enhancer.

Immunotherapy based approaches against c-MET are still subject of investigation. A novel dual inhibitor of MET and PD-1 has been designed and tested in several cancer models by Sun et al. [77]. It has shown strong anti-proliferative and anti-metastatic effects in vitro and in vivo and also reduced the production of inflammatory cytokines such as IL-6 and TNF-α thus suggesting it to be an important therapeutic agent against cancer progression. c-MET based immunotherapy has also been tested in pre-clinical mesothelioma models on the basis of MET chimeric antigen receptor T-cell (CAR-T) immunotherapy. CAR-T based immunotherapies are smart innovations that modify the genetic aspect of T-cells to direct their cytotoxic effects against cancer cells. Thayaparan and his team have engineered T-cells to express HGF as a chimeric antigen receptor to target c-MET expressing cancer cells [78]. This inhibited the cancer cell growth and metastasis along with the release of IFN-y and IL (interleukin)-2 from c-MET targeted CAR-T cells. The clinical evidences have been obtained from breast cancer patients, where after intra tumor injection, tumors displayed necrosis, loss of c-MET and infiltration of macrophages. Thus, these evidences suggest c-MET to be a good target to be used in respect to CAR-T cell therapy in cancer patients. However, c-MET based immunotherapies has to be till explored in HNSCC to explore its treatment efficacy against HNSCC tumor growth.

### c-MET signaling alterations in HNSCC

HGF is a major scatter factor (SF) protein that stimulates morphogenesis and motogenesis of epithelial cells in various organs [79]. In HNSCC, HGF is majorly secreted via tumor-associated fibroblasts (TAFs) in the tumor microenvironment [80, 81]. HGF is the primary ligand for c-MET activation in HNSCC. Increased expression of c-MET signaling takes place due to variety of genetic abnormalities including MET mutations and amplification of the MET gene [82]. MET mutations are frequently observed in the MET tyrosine kinase domain, sema and juxtamembrane domains in HNSCC patient tumors. Most of the HNSCC patients showed a c-MET mutation profile of Y1235D which was detected in higher incidence [83]. c-MET can be activated mainly through three mechanisms, ligand binding which triggers dimerization and transactivation of the receptor. c-MET is overexpressed in 78% of HNSCC cases having phosphorylated at active sites Y1230, Y1234 and Y1235 [84, 85]. Aberrant c-MET signaling promotes tumor progression and enables the development of distant metastasis by increasing the invasive capacity of HNSCC tumor cells. This signaling pathway also stimulates the morphogenesis of epithelial cells to acquire this aggressive metastatic phenotype via epithelial-mesenchymal transition (EMT). Secondly, physical modifications in the receptor can also constitutively activate c-MET receptor through somatic mutations. Mutations in the kinase domain involving Y123D and Y1230C are majorly involved in the activation of the receptor in HNSCC [86]. Gain in MET copy number is present in almost 16% of the HNSCC patients and associated with an increased c-MET expression and poorer outcome [87]. Y1230C and Y1235D mutations consistently promote HNSCC metastasis. Y1235D mutation is also responsible for impaired local HNSCC tumor control, disrupts the radiation therapy and worsens recurrence.

HNSCC has been categorized by a common feature of lymph node invasion and highly predictive of patient mortality. Lymph node metastasis in HNSCC involves high c-MET expression although, MET gene amplification is low [88]. c-MET is expressed in all stages, with having high expression in N2 and N3 nodal metastasis [89]. c-MET staining positivity was significantly correlated with regional lymph node metastasis in HNSCC. High expression of HGF was observed in HNSCC patients as compared to the healthy individuals. At later stages of cancer primary tumor cells metastasize to neighbor tissues as well as organs or lymph nodes developing into secondary tumors. Increased HGF expression has been correlated to anoikis resistance by fibronectin signaling in HNSCC by disrupting the integrin signaling [90].

c-MET pathway has been reported to be associated with several other signaling pathways in HNSCC. Wnt/β-Catenin pathway has been recognized as one of the important pathways responsible for pattern formation during embryogenesis, and also has prominent role in cancer [91]. β-Catenin dependent transcription takes place via c-MET in colon cancer cells, converting it into a cancer stem cell (CSC). CSC in HNSCC were inhibited with PF-2341066 (a c-MET inhibitor) and β-Catenin was shown to be the downregulating factor contributing to CSC elimination [92]. The Wnt pathway frizzled class receptor 8 (FZD8) expression rescued impaired HNSCC cells that were treated with c-MET inhibitor [92]. It was also noted by the same group that FZD8 was upregulated by c-MET signaling through ERK/c-Fos cascade. C-Src is yet another pathway that control various biological functions where its aberrant regulation can lead to anchorage-independent growth, survival, tumor vascularity, migration, metastasis, survival and invasion [93]. Activated c-Src mediates erlotinib resistance in HNSCC by stimulating c-MET independent of ligand [94]. Sen et al. earlier showed in both xenograft and in vitro models that combination inhibition of c-MET and c-Src resulted in synergistic cytotoxicity, enhanced apoptosis, and decreased tumor size [95]. Bhowmick et al. showed that transforming growth factor (TGF)-β type II receptor knockout in mice gave rise to prostate and gut epithelial tumors by activating c-MET through paracrine overexpression of HGF by stromal cells [96]. TGF-β is mediated by the transcription factor mothers against decapentaplegic homology (SMAD) that bind SMAD binding element (SBE) of target genes, regulating their expression [97]. SMAD deletion caused HGF upregulation, contributing to angiogenesis in mice [98]. The HGF promoter of keratinocytes have one such SBE that allows binding of SMAD 1, 2, and 4 families. In HNSCC, in vitro knockdown of SMAD 4 induced cetuximab resistance by activating TGF-β and c-MET pathways [99]. EGFR and c-MET have in common similar downstream pathways: MAPK (RAS/Rapidly accelerating fibrosarcoma (RAF)/ MAP kinase kinases (MEK)/ERK) and PI3K/AKT/mechanistic target of rapamycin (mTOR). To eliminate these aberrant pathway modalities altogether, dual EGFR/c-MET blockade is of interest. Seiwert et al. showed that dual blockade of SU11274 (c-MET inhibitor) and erlotinib (EGFR inhibitor) in HNSCC lines produce greater-than-additive inhibition of cell growth via erbB3/AKT signaling [86]. Further, Lieu et al. demonstrated that cell lines treated with foretinib and erlotinib or lapatinib [EGFR/human epidermal growth factor (HER) 2 inhibitor] synergistically inhibit HNSCC growth [100]. Even more, crizotinib (c-MET inhibitor) and gefitinib given in combination potentiated the effects of cell line invasion, wound healing, and proliferation; increased antitumor activity in vivo when compared to EGFR inhibition alone was shown by Guo et al. They also noticed that in the absence of HGF, TGF-α (an EGFR ligand) activates c-MET when EGFR is blocked [101]. The intertwining of the RTK’s c-MET and EGFR may explain acquired or constitutive EGFR resistance. c-MET/EGFR co-expression is common. It is likely that c-MET/HGF expression is a common mechanism of EGFR treatment resistance in HNSCC [102]. Wheeler et al. further show that cell lines resistance to cetuximab highly express c-MET, EGFR, HER-2, and HER-3 [103].

### c-MET/HGF signaling in HNSCC metabolism

The c-MET/HGF signaling is also known to contribute widely in metabolic reprogramming of tumor cells. Increased glucose metabolism is highly preferred by cancer cells to yield much higher ATP and it also generates biosynthesis relevant precursor molecules. This phenomenon was first described by Otto Warburg and is known as Warburg phenomenon [104]. HGF has been reported to enhance the expression of glucose transporters GLUT-1 and GLUT-4 in several types of cancers. Although, high glucose consumption and metabolism in head and neck cancer is clinically relevant, since the uptake of (18)F-2-fluro-2-deoxy-D-glucose (FDG) has been significantly observed through PET/CT scans. Kumar et al. has reported an interesting study on HGF related upregulation of glycolysis in HNSCC cells [81]. The study has also been associated with the upregulation of key glycolytic genes such as HK2, PFK1, and MCT1 in HGF stimulated HNSCC cell lines. Verena et al. provided further evidence that HGF/c-MET signaling plays an important role in maintaining a central hallmark of cancer, the Warburg effect [105]. This area is yet to be explored in head and neck cancer progression to find suitable targets against head and neck cancer progression.

### c-MET/HGF signaling in tumor microenvironment

Over ages, scientists have been focusing on the core of the cancer tissue to find evidences for cancer treatment. Although several studies have been focusing on other aspects of the tumor such as tumor microenvironment (TME) to find evidences for the progression of the disease. The tumor microenvironment (TME) is a complex tissue structure composed of fibroblasts, blood vessels, immune cells and the extracellular matrix. The TME surrounded through the cancer cells help in the development, progression, drug resistance and metastasis. Several studies have suggested the paracrine activation of c-MET/HGF signaling in HNSCC [81]. Fibroblasts co-cultured with tumor cells secrete high amount of HGF as compared to fibroblasts cultured in the absence of tumor cells. Overexpression of HGF has been reported in the tumor microenvironment of 50% of the HNSCC patient samples [105]. Also, studies with ficlatuzumab have demonstrated its ability to reduce the effects of TAFs on proliferation, migration, and invasion in HNSCC [106]. Also, HGF derived from TME has reported to increase radioresistance and chemoresistance in several cancers. Although, very less evidences of radioresistance is reported in HNSCC. There has been increased studies between immune cells in TME and cancer cells. Innate and adaptive immunity play important roles in suppressing or promoting tumorigenesis. The increased lactate levels due to increased glycolytic flux in HNSCC has shown to potentially suppress the proliferation activity of human CTLs [107]. HGF mediated upregulation of PDL-1 was shown to be associated with PI3K signaling pathway which is frequently mutated in HNSCC. PD-1 is being highly investigated to be a therapeutic target against HNSCC progression. The prominent role of c-MET/HGF signaling on immune surveillance and activation requires further study in HNSCC to develop novel therapeutic target against HNSCC progression.

### c-MET and chemoresistance in HNSCC

**Fig. 2:**
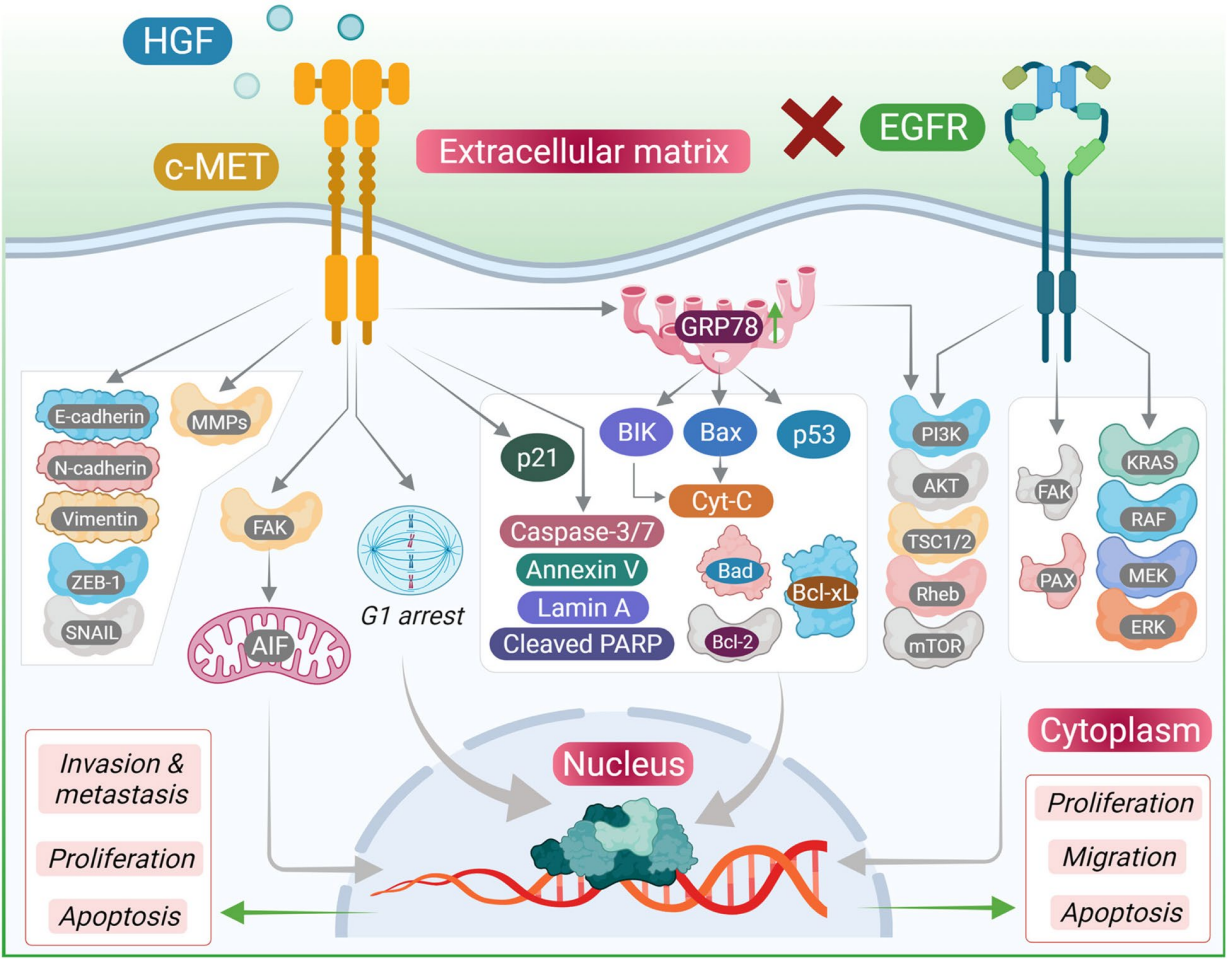
Chemotherapy resistant acquired in cancer cells via altered MET signaling. This includes overexpression of MET, activation sustained by HGF secreted by CAFs (cancer associated fibroblasts), constitutive activation of MET and secretion of HGF which is not normally expressed by epithelial cells, leading to an autocrine activation loop. This diagram illustrates the effect of EGFR inhibition through monoclonal antibodies alter/ activate MET signaling in cancer cells and causes the chemoresistance in cancer cells through c-MET activation which simultaneously cause reduced apoptosis, increased proliferation, enhanced DNA repair, upregulation of drug efflux and stimulation of epithelial-mesenchymal transition. All these changes contribute to the development of chemotherapy resistance

Chemoresistance has been a topic of concern ever since cancer cells have found their own ways to escape the inhibitory effects of chemodrugs. In a similar concern, therapies targeting EGFR has found resistance to the treatment due to the activation of c-MET signaling axis **(**Fig. 2**)** [108].

EGFR is a similar tyrosine kinase receptor as c-MET has been reported to be highly expressed in 90% of the HNSCC patients. Highly expressed EGFR contributes towards regional lymph node metastasis and poor outcomes. Chemoresistance towards EGFR inhibition by small molecule tyrosine kinase inhibitors such as gefitinib or erlotinib are common features in clinical trials. Upregulation of c-MET has also reported its contribution towards cetuximab resistance in HNSCC patients [109]. EGFR resistance has been rarely correlated towards its mutation profile rather has been largely correlated with activation of other receptor tyrosine kinases. Studies focusing on c-MET inhibition has reported EGFR sensitivity towards erlotinib drug as well as erlotinib sensitization in a dominant-active c-Src subset of EGFR overexpressing HNSCC lines [94].

### c-MET based targeted therapy

**Fig. 3:**
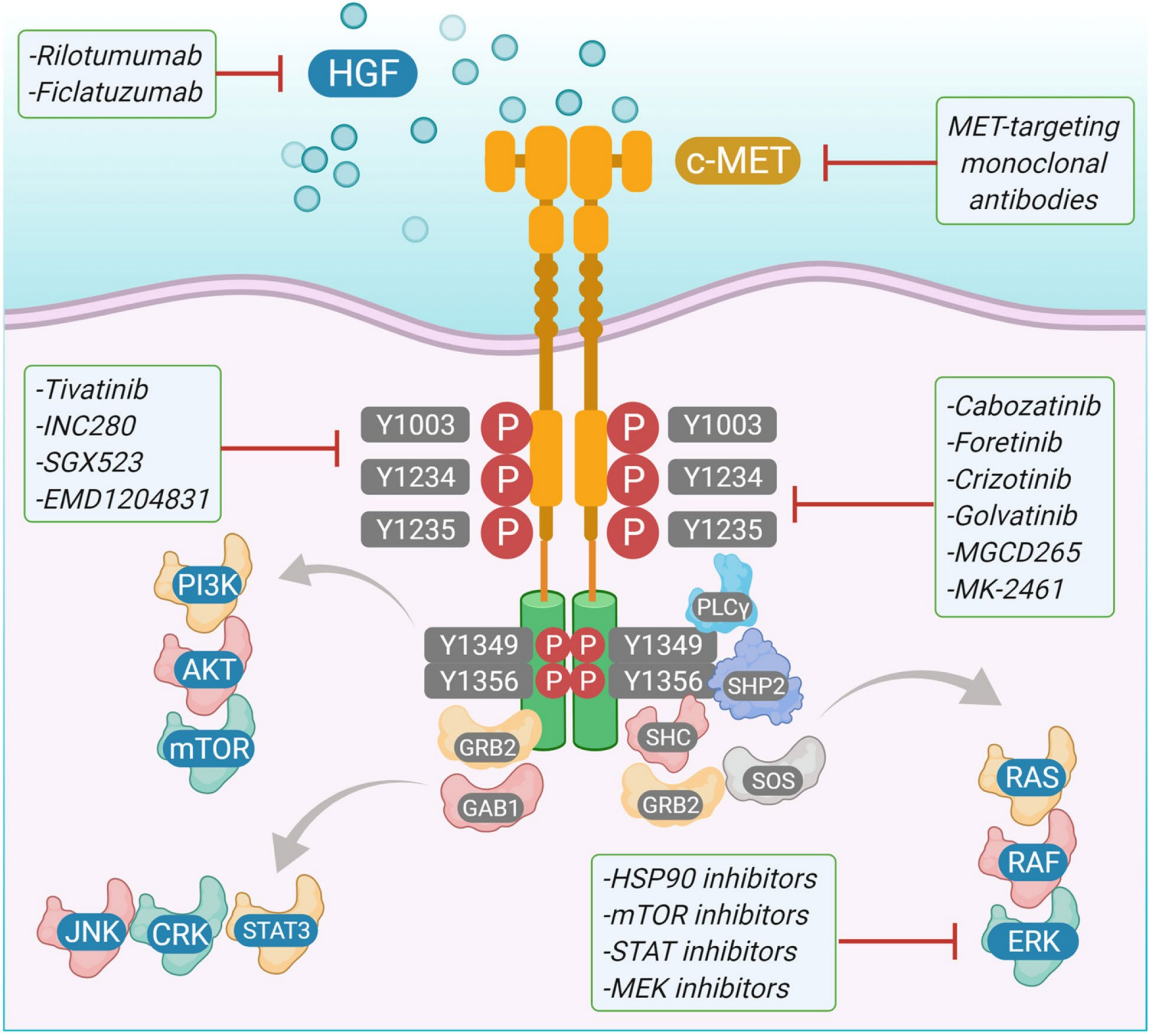
MET therapeutic strategies in the inhibition of cancer survival. Aberrant HGF stimulation of MET in human cancer through paracrine signaling activates the receptor. MET activation results in the recruitment and activation of downstream adaptor proteins and kinase targets resulting in a multitude of effects such as increased cell proliferation, cell cycle progression, scattering, motility, survival, extracellular matrix remodeling, and changes in metabolism. Therapeutic intervention strategies to block and inhibit MET receptor oncogenic signaling cascade include blocking ligand-receptor interaction, preventing receptor dimerization, blocking MET kinase intrinsic activity, and inhibiting specific downstream signal transducers

c-MET has a major contribution towards compensating for inhibition of RTK pathways that help in proliferation and metastasis in HNSCC. Therefore, targeting c-MET along with other receptor tyrosine kinases can bring effective therapeutic strategies. There are few c-MET inhibitors already in use for combination therapies against HNSCC **(**Fig. 3**) (**Table 1**)**.

**Table 1:** List of c-Met inhibitors

Drug	Study	Phase
Crizotinib (PF0234I066)	NCT00585I95	I
Cabozantinib (XL184)	NCT0I8664I0	II
	NCT0I708954	II
	NCT00940225	II
Foretinib (GSKI363089)	NCT0I068587	I/II
Tepotinib (EMDI2I4063)	NCT02864992	II
Rilotumumab (AMG102)	NCT02137343	III
Ficlatuzumab	NCT03422536	II
Tivantinib (ARQ197)	NCT01244191	III
Capmatinib (INC280, INCB28060)	NCT0I324479	I
	NCT0I60336	II

Crizotinib, a class Ia small molecule dual inhibitor of c-MET and anaplastic lymphoma kinase (ALK) had been proposed for advanced non-small cell lung cancer therapy with activation of either c-ros oncogene 1 (ROS-1) or ALK [110]. Crizotinib shows its inhibitory effects towards c-MET and ALK by binding to the activation loop and stabilizing the autoinhibitory conformation of each kinase. Pre-clinical studies involving crizotinib (IC50 4.1–4.7uM) showed to be effective against HNSCC cell lines as well as had a significant inhibition of both wound-closure and invasion [111]. Crizotinib induced apoptosis and greatly decreased the tumor burden in a patient derived xenograft in vivo model. In combination with the EGFR inhibitor gefitinib, crizotinib showed greater inhibition of cell survival in vitro as well as impaired wound closure and invasion. Similar results were obtained in vivo with enhanced reduction of patient xenograft tumor volume and the number of cells positive for proliferation markers. Conversely, when crizotinib was combined with radiation therapy, inhibition of c-MET appeared to reduce radiosensitivity in vitro as measured by colony forming assay and in vivo using a mouse model [112]. To date, no clinical trials have been performed using crizotinib to treat HNSCC. Capmatinib is a class Ib small molecule inhibitor against c-MET that has been used in clinical trials. It shows its action by interacting with the autoinhibitory loop of c-MET as well as take advantage of small ATP binding site of c-MET to have greater specificity against the kinase molecule [113]. Unfortunately, little in vitro data is available for the effects of capmatinib on head and neck cell lines; however, it is known to be a potent inhibitor of c-MET in a kinase assay (enzymatic IC50 0.13 nM) and proliferation of other tumor lines (cell IC50 1.2–12.4 nM) [114]. In tumor-bearing mice, capmatinib was able to inhibit tumor growth and even cause complete regression of some tumor lines without any noticeable toxicity. A study determining safety and tolerability of capmatinib in advanced refractory c-MET-dependent solid tumors (NCT01324479) showed well toleration and showed anti-tumor activity and has been recommended now in tablet formulation. Another Phase Ib/II trial exploring the safety and efficacy of capmatinib treatment with cetuximab, an anti-EGFR antibody, in HNSCC and metastatic colorectal cancers positive for c-MET has recently been suspended for unknown reasons (NCT02205398). Golvatinib is another class II c-MET inhibitor used in clinical trials and are large hydrophobic molecules that binds to the back pocket of c-MET and are usually less specific [115]. It is specifically used against c-MET as well as vascular endothelial growth factor (VEGFR)-2. In preclinical studies, it inhibited proliferation of a broad range of tumor cell lines (cell IC50 6.2 nM to 4.3 μM) and inhibited HUVEC growth in response to HGF and VEGF as an in vitro model of angiogenesis [116]. Golvatinib also greatly reduced tumor burden in mouse xenograft models and blood vessel density in remaining tumors. Golvatinib has been in clinical trials against HNSCC. The first study was based on Phase I/II trial of EGFR inhibition with cetuximab along with or without golvatinib (NCT01332266).

The second clinical trial was meant to determine the efficacy of golvatinib treatment in conjunction with capecitabine and cisplatin (NCT01355302). Unfortunately, this trial did not pass Phase I with five of the seven enrolled patients experiencing serious adverse effects, including supraventricular tachycardia, convulsion, and pulmonary embolism. Foretinib is a class II inhibitor targeting c-MET, VEGFR2, Recepteurd’ Origine Nantais (RON), kinase insert domain receptor (KDR), and Fms related tyrosine kinase 1 (Flt-1) with potent inhibition of c-MET kinase activity (enzymatic IC50 1.16 nM) [100]. In HNSCC cell lines, foretinib alone strongly inhibited cell growth (cell IC50 0.61–0.79 μM) and exhibited synergistic inhibition of cell proliferation when combined with the EGFR inhibitor erlotinib [117]. Foretinib has also recently been demonstrated to contribute to radiosensitization in models of esophageal squamous cell carcinoma. Following irradiation, foretinib-treated cells had diminished repair of DNA double-stranded breaks and underwent more apoptosis. This effect was confirmed in vivo as seen by reduced tumor burden in mouse xenografts. It is unclear why inhibition of c-MET by foretinib increased radiosensitivity, whereas inhibition with crizotinib appeared to have the opposite effect, though this phenomenon is likely due to other kinases targeted by these two molecules. A clinical trial examining the safety and efficacy of foretinib alone in treating recurrent, metastatic HNSCC was terminated after Phase I after none of the enrolled patients presented partial or complete response (NCT00725764). Though this trial did not progress, enrolled patients had a 50% disease stabilization rate, and 6 of the 14 patients showed shrinking tumor size. No clinical trials to date have used foretinib in combination with other agents to treat HNSCC. Ficlatuzimab is currently the only biological therapy undergoing trial that targets the c-MET/HGF axis in HNSCC. It is a humanized monoclonal antibody that sequesters HGF, thereby preventing the stimulation of c-MET by HGF. Given its mechanism of action, however, it is important to recognize that ficlatuzumab is unable to inhibit HGF-independent c-MET activation [106]. Ficlatuzumab has been demonstrated to interfere with stromal contributions to proliferation, migration, and invasion in a preclinical in vitro model of HNSCC, as well as epithelial to mesenchymal transition (EMT) [118]. Ficlatuzumab is currently being tested in combination with cetuximab in a Phase Ib clinical trial of recurrent and metastatic HNSCC (NCT02277197).

Considering c-MET in aspects of cell signaling network through cross-talking with EGFR, VEGFR-2 and other signaling molecules, it is necessary to develop antibodies that are able to inhibit simultaneously two signaling cascades to achieve maximal therapeutic activity [77, 119]. Therapeutics such as bispecific therapeutic monoclonal antibodies (TMAMBs) targeting c-MET has been validated in clinical studies. The use of a bispecific antibody to simultaneously activate the T-cell immune response upon inhibition of MET signaling is also an attractive strategy. Currently, EGFR, epithelial cell adhesion molecule (EpCAM), VEGFR-2, and programed death-1 (PD-1) have been selected for development of MET-based bispecific TMABs.

## Conclusion

The incidence of HNSCC continues to rise and is predicted to rise by 30% by 2030. HNSCC has been characterized by genetic instability with frequent loss or gain of chromosomal regions. In addition to genetic alterations, epigenetic changes also have a role in driving HNSCC oncogenesis. Multiple pathways and processes contribute to the invasion and metastasis of HNSCC tumor cells. c-MET has been an important target against HNSCC because of its involvement in metabolic dysregulation, tumor-microenvirment and immune modulation. Dysregulation in HGF/c-MET signaling leading to uncontrolled proliferation, motility, invasiveness, and angiogenesis can play an essential role in the development, progression, and survival of cancer including HNSCC. Its association with several other major downstream pathways contributing towards cancer proliferation and metastasis has made it to be explored more and find suitable targets against it. Importantly, c-MET has also been associated with chemoresistance by bypassing traditionally clinically inhibited signals such as EGF. Gefitinib along with c-MET inhibited demonstrated additive synergetic effect in cell line models. Numerous investigations on c-MET aberrant signaling provide a strong rationale for exploring the therapeutic potential of specific receptor antagonists. Consequently, a crucial task will be to select the more active agents and integrate them effectively in well-designed clinical trials incorporating predictive biomarkers such as gene mutations, amplifications, and immunohistochemical c-MET overexpression. The complex crosstalk in cellular control networks opens new avenues for combination treatment protocols involving EGFR inhibitors and possibly also other targeted agents. There are abundant possibilities for bringing c-MET inhibitors to the clinic as a targeted therapeutic drugs fostering checks through metabolic signaling, tumor-microenvironment and immune modulations in HNSCC. 
